# Triaqua­chlorido(18-crown-6)barium chloride

**DOI:** 10.1107/S1600536812004990

**Published:** 2012-02-10

**Authors:** Min-Min Zhao

**Affiliations:** a Ordered Matter Science Research Center, College of Chemistry and Chemical Engineering, Southeast University, Nanjing 210096, People’s Republic of China

## Abstract

In the title compound, [BaCl(C_12_H_24_O_6_)(H_2_O)_3_]Cl, the Ba^II^ atom, the coordinating and free Cl^−^ anions, one coordinating water mol­ecule and two O atoms of an 18-crown-6 mol­ecule lie on a mirror plane. The environment of the ten-coordinate Ba^2+^ ion is defined by one Cl atom, three water mol­ecules and six O atoms from the macrocyclic ether. The macrocycle adopts a conformation with an approximate *D*_3*d*_ symmetry. In the crystal, O—H⋯Cl hydrogen bonds link the complex cations and Cl^−^ anions into a two-dimensional network parallel to (010). An intra­molecular O—H⋯Cl hydrogen bond is also present.

## Related literature

For the properties and structures of related compounds, see: Fu *et al.* (2007 ▶, 2008 ▶, 2009 ▶); Fu & Xiong (2008 ▶). For the ferroelectric properties of related derivatives, see: Fu *et al.* (2011*a* ▶,*b* ▶); Fu, Zhang, Cai, Ge *et al.* (2011 ▶).
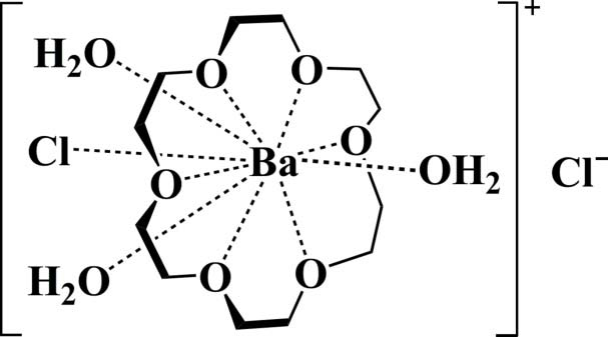


## Experimental

### 

#### Crystal data

[BaCl(C_12_H_24_O_6_)(H_2_O)_3_]Cl
*M* *_r_* = 526.59
Orthorhombic, 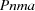

*a* = 14.962 (3) Å
*b* = 13.416 (3) Å
*c* = 10.347 (2) Å
*V* = 2077.0 (7) Å^3^
*Z* = 4
Mo *K*α radiation
μ = 2.21 mm^−1^
*T* = 293 K
0.30 × 0.25 × 0.15 mm

#### Data collection

Rigaku Mercury2 CCD diffractometer
Absorption correction: multi-scan (*CrystalClear*; Rigaku, 2005 ▶) *T*_min_ = 0.90, *T*_max_ = 1.00
20436 measured reflections
2478 independent reflections
2284 reflections with *I* > 2σ(*I*)
*R*_int_ = 0.038

#### Refinement

*R*[*F* ^2^ > 2σ(*F* ^2^)] = 0.026
*wR*(*F* ^2^) = 0.047
*S* = 1.20
2478 reflections
118 parameters
4 restraints
H-atom parameters constrained
Δρ_max_ = 0.49 e Å^−3^
Δρ_min_ = −0.80 e Å^−3^

### 

Data collection: *CrystalClear* (Rigaku, 2005 ▶); cell refinement: *CrystalClear*; data reduction: *CrystalClear*; program(s) used to solve structure: *SHELXTL* (Sheldrick, 2008 ▶); program(s) used to refine structure: *SHELXTL*; molecular graphics: *XP* in *SHELXTL* and *DIAMOND* (Brandenburg, 1999 ▶); software used to prepare material for publication: *SHELXTL*.

## Supplementary Material

Crystal structure: contains datablock(s) I, global. DOI: 10.1107/S1600536812004990/hy2512sup1.cif

Structure factors: contains datablock(s) I. DOI: 10.1107/S1600536812004990/hy2512Isup2.hkl

Additional supplementary materials:  crystallographic information; 3D view; checkCIF report

## Figures and Tables

**Table 1:** Hydrogen-bond geometry (Å, °)

*D*—H⋯*A*	*D*—H	H⋯*A*	*D*⋯*A*	*D*—H⋯*A*
O1*W*—H1*WA*⋯Cl1^i^	0.82	2.66	3.450 (3)	161
O1*W*—H1*WB*⋯Cl2^ii^	0.82	2.41	3.216 (3)	168
O2*W*—H2*WA*⋯Cl1	0.82	2.38	3.180 (2)	165
O2*W*—H2*WB*⋯Cl2	0.82	2.44	3.144 (2)	145

Symmetry codes: (i) 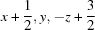; (ii) 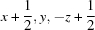.

**Fig. 1.**
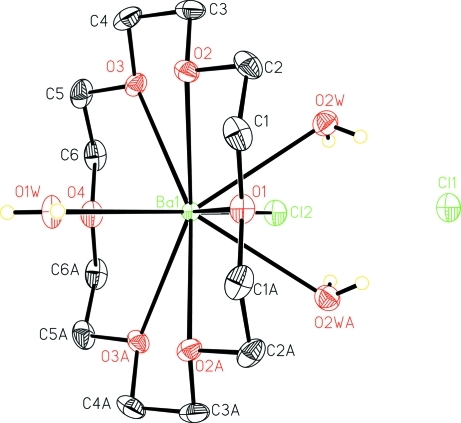
Molecular structure of the title compound. Displacement ellipsoids are drawn at the 30% probability level. H atoms attached to C atoms have been omitted for clarity. [Symmetry code: (A) x, 1/2-y, z.]

**Fig. 2.**
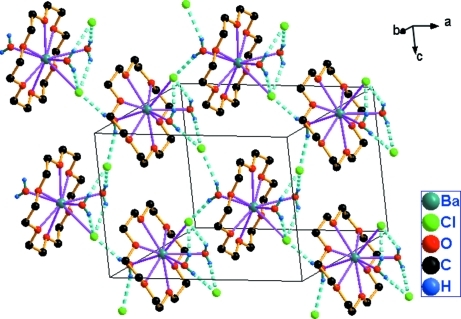
The crystal packing of the title compound, showing hydrogen-bonding interactions (dashed lines). H atoms not involved in hydrogen bonds have been omitted for clarity.

## supplementary crystallographic information

### Comment

Coordination compounds have attracted more attention as phase transition dielectric materials for their applications in memory storage (Fu *et al.*, 2007, 2008, 2009; Fu & Xiong 2008). With the purpose of obtaining phase transition crystals, various complexes have been studied and a series of new materials with organic and inorganic molecules have been elaborated (Fu *et al.*, 2011*a*,*b*; Fu, Zhang, Cai, Ge *et al.*, 2011). In this study, we describe the crystal structure of the title compound.

The title compound was composed of one macrocyclic 18-crown-6 ether, one Ba^II^ cation, three water molecules, one coordinated Cl^-^ anion and one uncoordinated Cl^-^ anion (Fig. 1). Six non-H atoms (Ba1, O1W, O1, O4, Cl1 and Cl2) and two H atoms (H1WA, H1WB) are located on a mirror plane. The ten-coordinated Ba^II^ environment is defined by one terminal Cl atom, three water molecules and six O atoms from the macrocyclic ether. The macrocycle adopts a conformation with an approximate D_3d_ symmetry, with all O—C—C—O torsion angles being *gauche* and alternating in sign and all C—O—C—C torsion angles being *trans*. The structure is stabilized by intermolecular O—H···Cl hydrogen bonds (Table 1). These hydrogen bonds link the ionic units into a two-dimensional network parallel to (0 1 0) (Fig. 2). O2W is involved in an intramolecular O2W—H2WB···Cl2 hydrogen bond.

### Experimental

Commercial 18-crown-6 (6 mmol), HCl (10 mmol) and BaCl_2_ (6 mmol) were dissolved in a water/EtOH (*v*/*v* 1:1) solution. The solvent was slowly evaporated in air, affording colourless block-shaped crystals of the title compound suitable for X-ray analysis.

The dielectric constant of the title compound as a function of temperature indicates that the permittivity is basically temperature-independent, suggesting that this compound should not be a real ferroelectrics or there may be no distinct phase transition occurred within the measured temperature range. Similarly, the dielectric constant as a function of temperature also goes smoothly below 400 K, and there is no dielectric anomaly observed (dielectric constant ranging from 5.5 to 7.1).

### Refinement

H atoms attached to C atoms were positioned geometrically and treated as riding, with C—H = 0.97 Å and with *U*_iso_(H) = 1.2*U*_eq_(C). H atoms of the water molecules were located from a difference Fourier map and refined as riding, with O—H = 0.82 Å and with *U*_iso_(H) = 1.5*U*_eq_(O).

### Crystal data

**Table:**

[BaCl(C_12_H_24_O_6_)(H_2_O)_3_]Cl	*F*(000) = 1056
*M**_r_* = 526.59	*D*_x_ = 1.684 Mg m^−^^3^
Orthorhombic, *P**n**m**a*	Mo *K*α radiation, λ = 0.71073 Å
Hall symbol: -P 2ac 2n	Cell parameters from 2478 reflections
*a* = 14.962 (3) Å	θ = 3.0–27.5°
*b* = 13.416 (3) Å	µ = 2.21 mm^−^^1^
*c* = 10.347 (2) Å	*T* = 293 K
*V* = 2077.0 (7) Å^3^	Block, colourless
*Z* = 4	0.30 × 0.25 × 0.15 mm

### Data collection

**Table:**

Rigaku Mercury2 CCD diffractometer	2478 independent reflections
Radiation source: fine-focus sealed tube	2284 reflections with *I* > 2σ(*I*)
Graphite monochromator	*R*_int_ = 0.038
Detector resolution: 13.6612 pixels mm^-1^	θ_max_ = 27.5°, θ_min_ = 3.0°
ω scans	*h* = −19→19
Absorption correction: multi-scan (*CrystalClear*; Rigaku, 2005)	*k* = −17→17
*T*_min_ = 0.90, *T*_max_ = 1.00	*l* = −13→13
20436 measured reflections	

### Refinement

**Table:**

Refinement on *F*^2^	Primary atom site location: structure-invariant direct methods
Least-squares matrix: full	Secondary atom site location: difference Fourier map
*R*[*F*^2^ > 2σ(*F*^2^)] = 0.026	Hydrogen site location: inferred from neighbouring sites
*wR*(*F*^2^) = 0.047	H-atom parameters constrained
*S* = 1.20	*w* = 1/[σ^2^(*F*_o_^2^) + (0.0112*P*)^2^ + 1.2653*P*] where *P* = (*F*_o_^2^ + 2*F*_c_^2^)/3
2478 reflections	(Δ/σ)_max_ < 0.001
118 parameters	Δρ_max_ = 0.49 e Å^−^^3^
4 restraints	Δρ_min_ = −0.80 e Å^−^^3^

### Special details

**Table:**

Geometry. All esds (except the esd in the dihedral angle between two l.s. planes) are estimated using the full covariance matrix. The cell esds are taken into account individually in the estimation of esds in distances, angles and torsion angles; correlations between esds in cell parameters are only used when they are defined by crystal symmetry. An approximate (isotropic) treatment of cell esds is used for estimating esds involving l.s. planes.
Refinement. Refinement of F^2^ against ALL reflections. The weighted R-factor wR and goodness of fit S are based on F^2^, conventional R-factors R are based on F, with F set to zero for negative F^2^. The threshold expression of F^2^ > 2sigma(F^2^) is used only for calculating R-factors(gt) etc. and is not relevant to the choice of reflections for refinement. R-factors based on F^2^ are statistically about twice as large as those based on F, and R- factors based on ALL data will be even larger.

### Fractional atomic coordinates and isotropic or equivalent isotropic displacement parameters (Å^2^)

**Table:**

	*x*	*y*	*z*	*U*_iso_*/*U*_eq_	
Ba1	0.330565 (12)	0.2500	0.419354 (17)	0.02669 (6)	
Cl2	0.17702 (6)	0.2500	0.19266 (9)	0.0506 (2)	
Cl1	0.07097 (6)	0.2500	0.68085 (10)	0.0498 (2)	
O2	0.35766 (12)	0.07191 (13)	0.56515 (16)	0.0417 (4)	
O3	0.38055 (12)	0.06918 (14)	0.29713 (17)	0.0443 (4)	
C1	0.33416 (19)	0.1618 (2)	0.7592 (2)	0.0520 (7)	
H1A	0.3012	0.1635	0.8398	0.062*	
H1B	0.3974	0.1581	0.7792	0.062*	
C5	0.4373 (2)	0.0747 (3)	0.1869 (3)	0.0627 (9)	
H5A	0.4323	0.0140	0.1365	0.075*	
H5B	0.4990	0.0819	0.2142	0.075*	
C3	0.3403 (2)	−0.01562 (19)	0.4902 (3)	0.0542 (7)	
H3A	0.2785	−0.0162	0.4621	0.065*	
H3B	0.3510	−0.0747	0.5419	0.065*	
C2	0.30708 (19)	0.0729 (2)	0.6825 (3)	0.0516 (7)	
H2A	0.3185	0.0126	0.7315	0.062*	
H2B	0.2437	0.0760	0.6631	0.062*	
C4	0.4007 (2)	−0.0152 (2)	0.3759 (3)	0.0574 (8)	
H4A	0.4625	−0.0120	0.4042	0.069*	
H4B	0.3926	−0.0760	0.3266	0.069*	
C6	0.4108 (2)	0.1612 (3)	0.1076 (3)	0.0597 (9)	
H6A	0.4462	0.1633	0.0291	0.072*	
H6B	0.3483	0.1556	0.0836	0.072*	
O1W	0.50755 (16)	0.2500	0.4986 (3)	0.0530 (7)	
H1WA	0.5090	0.2500	0.5778	0.079*	
H1WB	0.5557	0.2500	0.4606	0.079*	
O4	0.42471 (17)	0.2500	0.1809 (2)	0.0472 (7)	
O1	0.31620 (16)	0.2500	0.6863 (2)	0.0402 (6)	
O2W	0.16525 (13)	0.13030 (15)	0.45333 (19)	0.0556 (5)	
H2WA	0.1351	0.1514	0.5136	0.083*	
H2WB	0.1453	0.1510	0.3846	0.083*	

### Atomic displacement parameters (Å^2^)

**Table:**

	*U*^11^	*U*^22^	*U*^33^	*U*^12^	*U*^13^	*U*^23^
Ba1	0.02774 (10)	0.02935 (10)	0.02298 (9)	0.000	−0.00053 (8)	0.000
Cl2	0.0459 (5)	0.0653 (6)	0.0405 (5)	0.000	−0.0125 (4)	0.000
Cl1	0.0445 (5)	0.0589 (6)	0.0461 (5)	0.000	0.0038 (4)	0.000
O2	0.0435 (9)	0.0375 (10)	0.0443 (10)	−0.0053 (8)	−0.0036 (8)	0.0071 (8)
O3	0.0418 (10)	0.0440 (10)	0.0473 (11)	0.0074 (9)	−0.0011 (8)	−0.0128 (9)
C1	0.0523 (16)	0.075 (2)	0.0287 (12)	−0.0107 (16)	−0.0043 (13)	0.0145 (14)
C5	0.0509 (17)	0.074 (2)	0.063 (2)	0.0052 (16)	0.0121 (15)	−0.0312 (18)
C3	0.0643 (19)	0.0293 (13)	0.0690 (19)	−0.0036 (14)	−0.0141 (17)	0.0033 (13)
C2	0.0533 (16)	0.0567 (18)	0.0450 (16)	−0.0140 (14)	−0.0026 (13)	0.0209 (14)
C4	0.0602 (19)	0.0411 (16)	0.071 (2)	0.0181 (14)	−0.0164 (16)	−0.0152 (15)
C6	0.0512 (17)	0.094 (3)	0.0336 (15)	−0.0055 (17)	0.0081 (13)	−0.0204 (16)
O1W	0.0313 (13)	0.079 (2)	0.0484 (15)	0.000	0.0028 (12)	0.000
O4	0.0467 (15)	0.0683 (18)	0.0267 (13)	0.000	0.0027 (11)	0.000
O1	0.0423 (14)	0.0581 (16)	0.0203 (11)	0.000	−0.0024 (10)	0.000
O2W	0.0542 (12)	0.0543 (12)	0.0582 (12)	0.0080 (10)	0.0028 (10)	0.0049 (10)

### Geometric parameters (Å, º)

**Table:**

Ba1—O1	2.770 (2)	C5—H5A	0.9700
Ba1—O1W	2.772 (3)	C5—H5B	0.9700
Ba1—O3	2.8360 (18)	C3—C4	1.488 (4)
Ba1—O3^i^	2.8360 (18)	C3—H3A	0.9700
Ba1—O4	2.841 (2)	C3—H3B	0.9700
Ba1—O2^i^	2.8545 (17)	C2—H2A	0.9700
Ba1—O2	2.8545 (17)	C2—H2B	0.9700
Ba1—O2W	2.970 (2)	C4—H4A	0.9700
Ba1—O2W^i^	2.970 (2)	C4—H4B	0.9700
Ba1—Cl2	3.2831 (10)	C6—O4	1.427 (3)
O2—C2	1.431 (3)	C6—H6A	0.9700
O2—C3	1.431 (3)	C6—H6B	0.9700
O3—C5	1.424 (3)	O1W—H1WA	0.8201
O3—C4	1.427 (3)	O1W—H1WB	0.8201
C1—O1	1.429 (3)	O4—C6^i^	1.427 (3)
C1—C2	1.488 (4)	O1—C1^i^	1.429 (3)
C1—H1A	0.9700	O2W—H2WA	0.8200
C1—H1B	0.9700	O2W—H2WB	0.8200
C5—C6	1.476 (4)		
			
O1—Ba1—O1W	77.25 (7)	C4—O3—Ba1	118.64 (15)
O1—Ba1—O3	117.71 (4)	O1—C1—C2	109.3 (2)
O1W—Ba1—O3	83.10 (5)	O1—C1—H1A	109.8
O1—Ba1—O3^i^	117.71 (4)	C2—C1—H1A	109.8
O1W—Ba1—O3^i^	83.10 (5)	O1—C1—H1B	109.8
O3—Ba1—O3^i^	117.60 (8)	C2—C1—H1B	109.8
O1—Ba1—O4	154.72 (7)	H1A—C1—H1B	108.3
O1W—Ba1—O4	77.47 (8)	O3—C5—C6	109.0 (2)
O3—Ba1—O4	58.80 (4)	O3—C5—H5A	109.9
O3^i^—Ba1—O4	58.80 (4)	C6—C5—H5A	109.9
O1—Ba1—O2^i^	58.95 (4)	O3—C5—H5B	109.9
O1W—Ba1—O2^i^	73.03 (4)	C6—C5—H5B	109.9
O3—Ba1—O2^i^	156.08 (5)	H5A—C5—H5B	108.3
O3^i^—Ba1—O2^i^	58.82 (5)	O2—C3—C4	108.5 (2)
O4—Ba1—O2^i^	112.86 (4)	O2—C3—H3A	110.0
O1—Ba1—O2	58.95 (4)	C4—C3—H3A	110.0
O1W—Ba1—O2	73.03 (4)	O2—C3—H3B	110.0
O3—Ba1—O2	58.82 (5)	C4—C3—H3B	110.0
O3^i^—Ba1—O2	156.08 (5)	H3A—C3—H3B	108.4
O4—Ba1—O2	112.86 (4)	O2—C2—C1	108.4 (2)
O2^i^—Ba1—O2	113.65 (7)	O2—C2—H2A	110.0
O1—Ba1—O2W	79.48 (6)	C1—C2—H2A	110.0
O1W—Ba1—O2W	139.52 (5)	O2—C2—H2B	110.0
O3—Ba1—O2W	79.04 (5)	C1—C2—H2B	110.0
O3^i^—Ba1—O2W	137.30 (5)	H2A—C2—H2B	108.4
O4—Ba1—O2W	121.05 (6)	O3—C4—C3	109.2 (2)
O2^i^—Ba1—O2W	120.55 (5)	O3—C4—H4A	109.8
O2—Ba1—O2W	66.62 (5)	C3—C4—H4A	109.8
O1—Ba1—O2W^i^	79.48 (5)	O3—C4—H4B	109.8
O1W—Ba1—O2W^i^	139.52 (5)	C3—C4—H4B	109.8
O3—Ba1—O2W^i^	137.30 (5)	H4A—C4—H4B	108.3
O3^i^—Ba1—O2W^i^	79.04 (5)	O4—C6—C5	108.7 (2)
O4—Ba1—O2W^i^	121.05 (6)	O4—C6—H6A	109.9
O2^i^—Ba1—O2W^i^	66.62 (5)	C5—C6—H6A	109.9
O2—Ba1—O2W^i^	120.55 (5)	O4—C6—H6B	109.9
O2W—Ba1—O2W^i^	65.46 (8)	C5—C6—H6B	109.9
O1—Ba1—Cl2	131.15 (5)	H6A—C6—H6B	108.3
O1W—Ba1—Cl2	151.60 (6)	Ba1—O1W—H1WA	108.7
O3—Ba1—Cl2	82.30 (4)	Ba1—O1W—H1WB	134.1
O3^i^—Ba1—Cl2	82.30 (4)	H1WA—O1W—H1WB	117.1
O4—Ba1—Cl2	74.13 (6)	C6—O4—C6^i^	113.1 (3)
O2^i^—Ba1—Cl2	118.48 (4)	C6—O4—Ba1	112.90 (16)
O2—Ba1—Cl2	118.48 (4)	C6^i^—O4—Ba1	112.90 (16)
O2W—Ba1—Cl2	60.12 (4)	C1^i^—O1—C1	111.9 (3)
O2W^i^—Ba1—Cl2	60.12 (4)	C1^i^—O1—Ba1	120.76 (14)
C2—O2—C3	111.8 (2)	C1—O1—Ba1	120.76 (14)
C2—O2—Ba1	111.42 (15)	Ba1—O2W—H2WA	111.2
C3—O2—Ba1	112.00 (14)	Ba1—O2W—H2WB	91.0
C5—O3—C4	111.9 (2)	H2WA—O2W—H2WB	110.1
C5—O3—Ba1	118.02 (17)		
			
O1—Ba1—O2—C2	28.83 (15)	Ba1—O3—C4—C3	36.3 (3)
O1W—Ba1—O2—C2	113.93 (16)	O2—C3—C4—O3	−63.0 (3)
O3—Ba1—O2—C2	−153.90 (17)	O3—C5—C6—O4	63.5 (3)
O3^i^—Ba1—O2—C2	117.76 (18)	C5—C6—O4—C6^i^	173.19 (18)
O4—Ba1—O2—C2	−178.09 (15)	C5—C6—O4—Ba1	−57.1 (3)
O2^i^—Ba1—O2—C2	51.76 (17)	O1—Ba1—O4—C6	115.07 (19)
O2W—Ba1—O2—C2	−62.78 (15)	O1W—Ba1—O4—C6	115.07 (19)
O2W^i^—Ba1—O2—C2	−24.09 (16)	O3—Ba1—O4—C6	25.60 (18)
Cl2—Ba1—O2—C2	−94.34 (15)	O3^i^—Ba1—O4—C6	−155.5 (2)
O1—Ba1—O2—C3	154.96 (19)	O2^i^—Ba1—O4—C6	−179.65 (18)
O1W—Ba1—O2—C3	−119.95 (18)	O2—Ba1—O4—C6	49.8 (2)
O3—Ba1—O2—C3	−27.77 (16)	O2W—Ba1—O4—C6	−25.8 (2)
O3^i^—Ba1—O2—C3	−116.12 (19)	O2W^i^—Ba1—O4—C6	−104.06 (19)
O4—Ba1—O2—C3	−51.96 (18)	Cl2—Ba1—O4—C6	−64.93 (19)
O2^i^—Ba1—O2—C3	177.88 (14)	O1—Ba1—O4—C6^i^	−115.07 (19)
O2W—Ba1—O2—C3	63.35 (17)	O1W—Ba1—O4—C6^i^	−115.07 (19)
O2W^i^—Ba1—O2—C3	102.04 (17)	O3—Ba1—O4—C6^i^	155.5 (2)
Cl2—Ba1—O2—C3	31.79 (18)	O3^i^—Ba1—O4—C6^i^	−25.60 (18)
O1—Ba1—O3—C5	−143.25 (18)	O2^i^—Ba1—O4—C6^i^	−49.8 (2)
O1W—Ba1—O3—C5	−71.58 (18)	O2—Ba1—O4—C6^i^	179.65 (18)
O3^i^—Ba1—O3—C5	6.9 (2)	O2W—Ba1—O4—C6^i^	104.06 (19)
O4—Ba1—O3—C5	7.92 (18)	O2W^i^—Ba1—O4—C6^i^	25.8 (2)
O2^i^—Ba1—O3—C5	−67.9 (2)	Cl2—Ba1—O4—C6^i^	64.93 (19)
O2—Ba1—O3—C5	−145.89 (19)	C2—C1—O1—C1^i^	175.28 (16)
O2W—Ba1—O3—C5	144.92 (18)	C2—C1—O1—Ba1	−33.0 (3)
O2W^i^—Ba1—O3—C5	111.38 (18)	O1W—Ba1—O1—C1^i^	74.58 (19)
Cl2—Ba1—O3—C5	83.99 (18)	O3—Ba1—O1—C1^i^	149.64 (18)
O1—Ba1—O3—C4	−2.7 (2)	O3^i^—Ba1—O1—C1^i^	−0.5 (2)
O1W—Ba1—O3—C4	68.92 (19)	O4—Ba1—O1—C1^i^	74.58 (19)
O3^i^—Ba1—O3—C4	147.40 (16)	O2^i^—Ba1—O1—C1^i^	−3.11 (18)
O4—Ba1—O3—C4	148.4 (2)	O2—Ba1—O1—C1^i^	152.3 (2)
O2^i^—Ba1—O3—C4	72.6 (2)	O2W—Ba1—O1—C1^i^	−138.8 (2)
O2—Ba1—O3—C4	−5.39 (17)	O2W^i^—Ba1—O1—C1^i^	−72.05 (19)
O2W—Ba1—O3—C4	−74.58 (18)	Cl2—Ba1—O1—C1^i^	−105.42 (19)
O2W^i^—Ba1—O3—C4	−108.12 (18)	O1W—Ba1—O1—C1	−74.58 (19)
Cl2—Ba1—O3—C4	−135.51 (18)	O3—Ba1—O1—C1	0.5 (2)
C4—O3—C5—C6	178.0 (2)	O3^i^—Ba1—O1—C1	−149.64 (18)
Ba1—O3—C5—C6	−39.0 (3)	O4—Ba1—O1—C1	−74.58 (19)
C2—O2—C3—C4	−175.3 (2)	O2^i^—Ba1—O1—C1	−152.3 (2)
Ba1—O2—C3—C4	58.8 (2)	O2—Ba1—O1—C1	3.11 (18)
C3—O2—C2—C1	175.6 (2)	O2W—Ba1—O1—C1	72.05 (19)
Ba1—O2—C2—C1	−58.2 (2)	O2W^i^—Ba1—O1—C1	138.8 (2)
O1—C1—C2—O2	60.2 (3)	Cl2—Ba1—O1—C1	105.42 (19)
C5—O3—C4—C3	179.0 (2)		

Symmetry code: (i) *x*, −*y*+1/2, *z*.

### Hydrogen-bond geometry (Å, º)

**Table:**

*D*—H···*A*	*D*—H	H···*A*	*D*···*A*	*D*—H···*A*
O1*W*—H1*WA*···Cl1^ii^	0.82	2.66	3.450 (3)	161
O1*W*—H1*WB*···Cl2^iii^	0.82	2.41	3.216 (3)	168
O2*W*—H2*WA*···Cl1	0.82	2.38	3.180 (2)	165
O2*W*—H2*WB*···Cl2	0.82	2.44	3.144 (2)	145

Symmetry codes: (ii) *x*+1/2, *y*, −*z*+3/2; (iii) *x*+1/2, *y*, −*z*+1/2.
